# Supplementary material for: The interactome of intact mitochondria by cross-linking mass spectrometry provides evidence for coexisting respiratory supercomplexes
Source: Mol Cell Proteomics. 2017 Dec 8;17(2):216–32. doi: 10.1074/mcp.RA117.000470 (PMC5795388; doi:10.1074/mcp.RA117.000470)
Supplement: Supplemental Data [file supp_RA117.000470_133922_0_supp_23978_fzffwf.zip › spectra_annotation/mito_spectra_annotation/138-1-10-1-10-1.pdf]

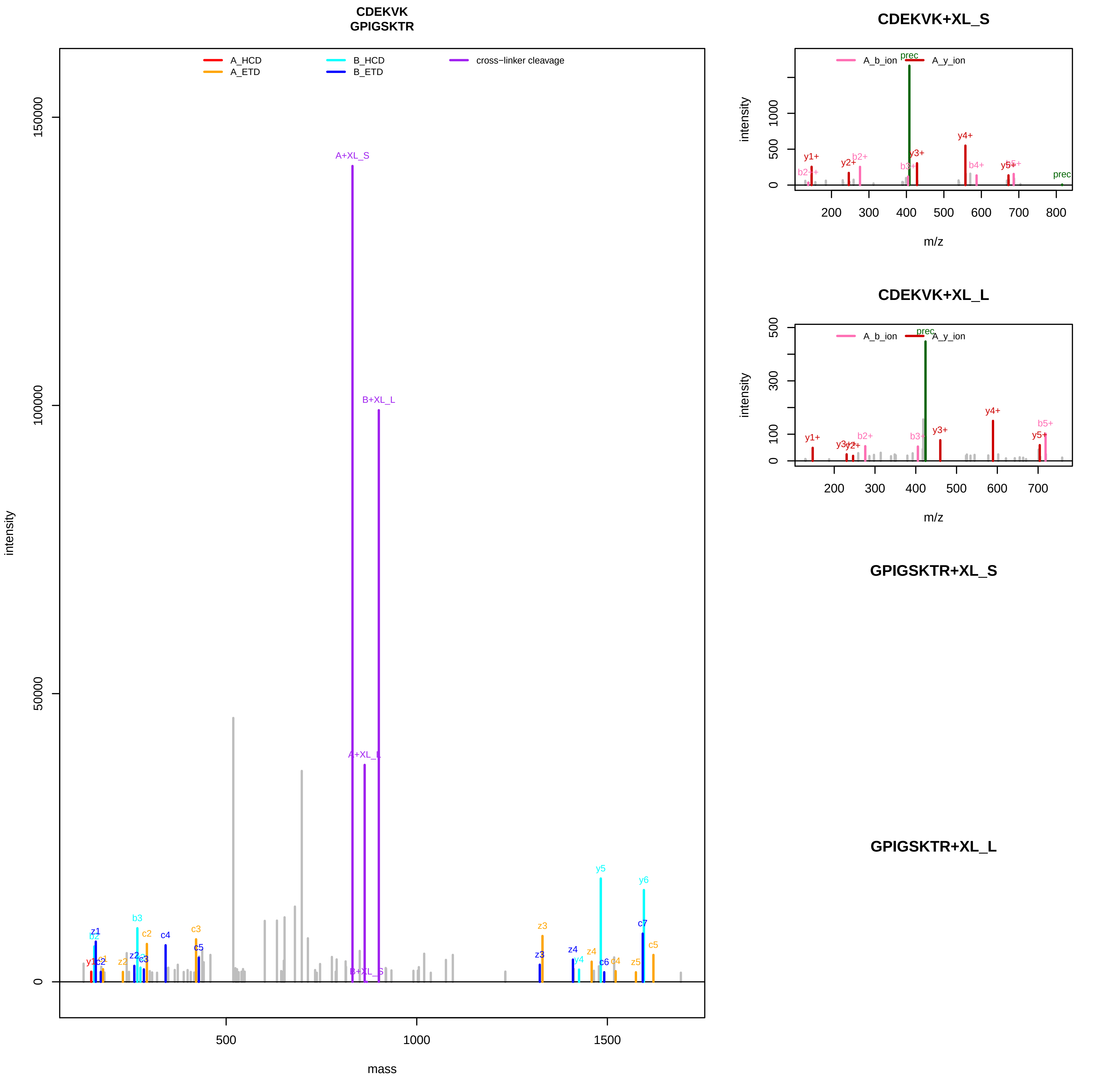
CDEKVK
GPIGSKTR
A_HCD
A_ETD
B_HCD
B_ETD
cross−linker cleavage
A+XL_S
B+XL_L
A+XL_L
B+XL_S
y5
y6
c7
z3
c3
b3
c4
c2
z1
c5
z4
y4
c6
c4
z5
c5
z2
y1
c2
z2
c3
0
50000
100000
150000
intensity
500
1000
1500
mass
CDEKVK+XL_S
A_b_ion
A_y_ion
prec
y1+
y2+
b2+
y3+
y4+
b4+
y5+
b5+
b3+
b2++
0
500
1000
200
300
400
500
600
700
800
m/z
intensity
CDEKVK+XL_L
A_b_ion
A_y_ion
prec
y1+
y3+
y2+
b2+
b3+
y3+
y4+
b5+
y5+
0
100
300
500
200
300
400
500
600
700
m/z
intensity
GPIGSKTR+XL_S
GPIGSKTR+XL_L
